# Etanercept improves pro-atherosclerotic biomarkers in children with juvenile idiopatic arthritis

**DOI:** 10.1186/1546-0096-9-S1-P169

**Published:** 2011-09-14

**Authors:** De Sanctis Sara, Breda Luciana, Del Torto Marianna, Nozzi Manuela, Lucantoni Marta, Sebastiani Marianna, Mohn Angelika, Chiarelli Francesco

## Background

There is large body of evidence on the correlation between chronic inflammatory diseases like rheumatoid arthritis (RA) and increased risk of cardiovascular diseases (CVD). Chronic inflammation is an independent risk factor for the development of early atherosclerosis. The possible role of anti TNF- α biologic drugs in reducing this risk arouses great interest among the scientific community. Many studies indicate the possibility that anti-TNF-α agents may reduce CVD risk and mortality in adult RA patients. There are only few data about the pediatric population.

## Aim

To determine the presence of early biomarkers of endothelial dysfunction in juvenile idiopathic arthritis (JIA) and to evaluate their improvement during anti TNF-α treatment with etanercept.

## Methods

We enrolled 30 children affected by JIA, all eligible for anti-TNF-α treatment. All patients were examined at baseline and after 6 months and 12 months of treatment with etanercept. Disease activity score for each patient was determined using JADAS (Juvenile Arthritis Disease Activity Score). Laboratory parameters included acute phase reactants (CRP and ESR), complete lipid profile, pro-inflammatory cytokines (TNF-α, IL-1β, IL-6, IFN-γ) and biomarkers of oxidative stress and vascular inflammation (8-iso-PGF_2_α, total nitric oxide, TXB_2_ and PGE_2_).

## Results

During the study all biomarkers of endothelial dysfunction progressively improved after 1 year of etanercept treatment in JIA children. At the same time all inflammatory parameters and JADAS scores significantly improved when compared to baseline. Pro-inflammatory cytokines showed significant reduction both after 6 and 12 months of treatment; at 6 months a relative increase in TNF-α and IL-6 was determined. Lipid profile, biomarkers of endothelial activation (total nitric oxide, PGE_2_, TXB_2_) and 8-iso-PGF_2α_ (a surrogate marker of oxidized LDL) showed significant improvement during treatment.

## Conclusion

We demonstrated beneficial effect of 1-year etanercept treatment on clinical disease activity, inflammatory indexes and oxidative biomarkers in JIA children. The role of inflammation on pre-atherosclerotic determinants justify the need of precocious interventions in JIA, in order to optimize the clinical outcome and to realize primary prevention of cardiovascular events in adulthood.

